# A mobile-based mental health improvement program for non-muscle invasive bladder cancer patients: Program development and feasibility protocol

**DOI:** 10.1192/j.eurpsy.2023.788

**Published:** 2023-07-19

**Authors:** Y. Kim, H. Lee, J. Park, S. Lee

**Affiliations:** 1 College of Nursing and Brain Korea 21 FOUR Project; 2 Mo-ImKim Nursing Research Institute and College of Nursing, Yonsei University, Seoul, Korea, Republic Of

## Abstract

**Introduction:**

Bladder cancer, which is primarily a non-muscle invasive bladder cancer (NMIBC), is prevalent worldwide and its incidence is increasing. NMIBC shows a high recurrence rate of 50-70%, and in 25% of cases, progresses to muscle-invasive disease (Saginala K *et al*. Med Sci 2020; 15) (Fernandez-Gomez, J *et al*. J Urol 2009; 182(5) 2195-2203). Frequent recurrence and consecutive medical interventions in patients with NMIBC lead to psychological problems such as anxiety, fear of recurrence, depression, and stress, resulting in reduced quality of life (Chung *et al*. Support Care Cancer 2019; 27(10), 3877-3885). It is expected that the increased accessibility and convenience of mobile health (mHealth) will be effective in providing a mobile-based psychological intervention program to promote the mental health of patients with NMIBC.

**Objectives:**

This study aims to develop a mobile-based mental health improvement program for NMIBC patients, design a protocol for evaluating feasibility, and provide preliminary evidence of the efficacy of the developed program.

**Methods:**

The program content was developed based on the results of a needs assessment conducted among patients with NMIBC through a cross-sectional study. The draft program was prepared by referring to the guidelines of the National Comprehensive Cancer Network and publications of the International Continence Society. Based on the developed draft, two professors of nursing, a professor of counseling psychology, a registered nurse, and a counseling practitioner verified the validity of the content before finalizing the program. The final version of the developed program consisted of one session on NMIBC knowledge and symptom management and five sessions on mental health improvement. Researchers sent an online link to the YouTube video comprising lecture materials and voice recordings of health professionals weekly using a mobile messenger (Kakao Talk) (Image 1). The topics of each session were as follows: Session 1 (Understanding of bladder cancer and treatment), Session 2 (Understanding and respecting myself ), Session 3 (Maintaining reasonable thoughts and positive emotions), Session 4 (Benefits of positive emotions and healthy communication), Session 5 (Living in a healthy way through stress management), and Session 6 (Finding happiness and meanings in daily life).

**Results:**

The protocol for evaluating the feasibility of the developed program is outlined in Image 2.

**Image:**

**Figure:**
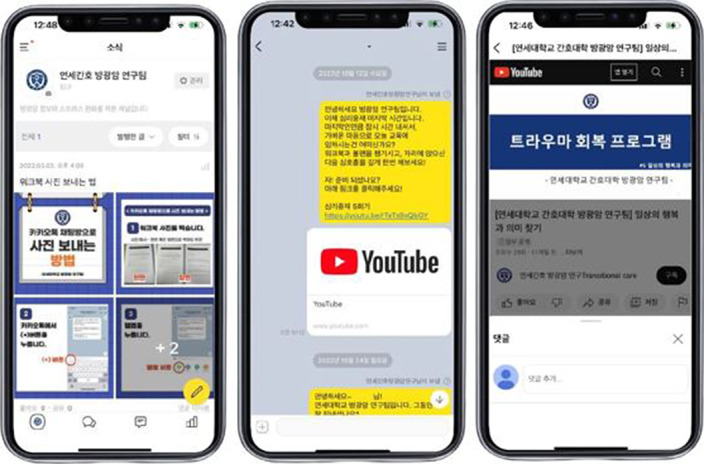

**Image 2:**

**Figure:**
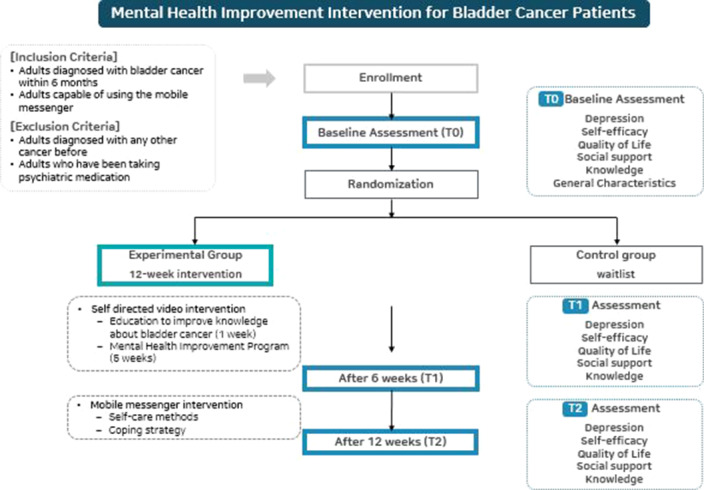

**Conclusions:**

The feasibility of the mental health improvement program for patients with NMIBC based on mobile messenger (KakaoTalk) will be evaluated through the developed protocol. Moreover, by introducing a program that reflects the feasibility of test results into practice, the results of this study can contribute to improving the quality of life of patients with NMIBC.

**Disclosure of Interest:**

None Declared

